# Resistin – 420 C/G polymorphism and serum resistin level in Iranian patients with gestational diabetes mellitus

**DOI:** 10.1186/s40200-015-0165-y

**Published:** 2015-04-28

**Authors:** Mohammad Ali Takhshid, Zinab Zare

**Affiliations:** Diagnostic Laboratory Sciences and Technology Research Center, School of Paramedical Sciences, Shiraz University of Medical Sciences, Shiraz, Iran

**Keywords:** Gestational diabetes, Resistin, Single nucleotide polymorphism- 420C/G

## Abstract

**Background:**

Resistin is a circulating adipokine with insulin-antagonizing effects. The aim of this study was to investigate the relationship between the single nucleotide polymorphism (SNP) -420C > G in the resistin gene with serum resistin levels, insulin resistance, and risk of gestational diabetes (GDM) in Iranian population.

**Method:**

75 GDM patients and 70 healthy pregnant women were enrolled in this study. Genotyping for SNP- 420C > G in the resistin gene was performed by the polymerase chain reaction- restriction fragment length polymorphism (PCR-RFLP) method. Serum resistin and insulin were measured by immunoassay. Blood glucose levels and lipid profile were measured by enzymatic methods. Homeostasis model of assessment for insulin resistance (HOMA-IR) were calculated.

**Result:**

GG genotype and G allele of SNP-420C > G were more frequent in GDM patients compared to non-GDM subjects. Serum resistin level was similar in GDM and non-GDM patients. The serum levels of resistin in GDM and non-GDM women with GG genotype were similar to those with GC + CC genotype. Multivariate logistic regression analysis after adjusting for confounding factors showed a higher susceptibility to GDM in patients with GG genotype compared to subjects with GG + GT genotype (odds ratio = 4.59, 95% CI; 1.96-10.71, p = 0.00). Serum resistin level was correlated with serum triglyceride, total and low density lipoprotein (LDL) cholesterol (p < 0.05) in GDM patients. No significant association was found between serum resistin, insulin resistance, and SNP-420C > G.

**Conclusion:**

The SNP-420C/G of resistin gene is associated with genetic susceptibility to GDM in our population. Further studies are necessary to confirm the role of this polymorphism in pathogenesis of GDM and to explore potential mechanisms by which it modulates susceptibility to GDM.

## Background

Gestational diabetes mellitus (GDM) is a type of glucose intolerance that occurs during pregnancy [1]. GDM and type 2 diabetes (T2D) are closely related disorders with a common pathogenesis [2]. Ethnicity, family history, dietary habit, physical inactivity and obesity are among risk factors implicated in susceptibility to both T2D and GDM [2,3]. Insulin resistance is an important characteristic of both T2D and GDM [2]. Several adipokines including leptin, visfatin, adiponectin and resistin are reported to be involved in development of insulin resistance [4,5].

Resistin is a cysteine-rich polypeptide with 108 amino acid residues synthesized and secreted by adiopcytes, immune and endothelial cells [6]. In pregnancy, the placenta is a major source of maternal circulating resistin [7]. Initial studies in rodent animal models suggested that resistin involves in insulin resistance and T2D. Indeed, up-regulation of resistin in obese animal rodent models, decrease in its circulating level by thiazolidinediones (TZDs), and decrease in plasma glucose level in response to anti-resistin antibody administration in obese animals have been reported [8]. However, the exact role of resistin in the pathogenesis of insulin resistance and T2D in humans is still unclear. Although the association between elevated circulating resistin and insulin resistance has been suggested in some studies [9,10], other studies fail to detect such an association [11,12]. Several inconsistent findings have also been reported from serum resistin levels in patients with GDM. Most of these findings showed no difference in the serum resistin level between GDM and normal glucose tolerant (NGT) subjects [13,14]. However, there are other reports indicating increased [15,16] or decreased [7] serum resistin concentrations in women with GDM compared with NGT pregnant women.

The gene encoded resistin (RETN gene) is located on 19p3.2 and composed of four exons and three introns. It is a polymorphic gene with several single nucleotide polymorphism (SNPs) in its promoter, introns and 3′-UTR (3′-untranslated region) regions [17]. Because of its possible influence on RETN gene expression and circulating resistin concentration, SNP-420C > G (rs 1862513) in the promoter is one of the most studied SNPs of RETN gene [17-20]. It has been reported that the promoter activity of RETN is higher in individuals with GG genotype of SNP-420C > G compared to CC/GC genotype [19]. The association of SNP-420C > G genotype with T2D has been shown in some previous studies [19,21]. Because of the close relationship between T2D and GDM, it is reasonable to evaluate the association between this SNP and susceptibility to GDM. Thus, the first aim of our study was to evaluate the association of SNP- 420C > G with risk of GDM in Iranian population. We also aimed to compare serum resistin level between GDM and non-GDM subjects, as well as its association with genotypes of SNP-420C > G. Furthermore, we examined possible associations between serum resistin levels with obesity, insulin resistance and serum biochemical abnormalities related to GDM.

## Material

All subjects enrolled in the present study were Iranian and recruited from outpatient clinic of Hafez and Zinabieh Hospitals in Shiraz, Iran, between September 2011 and May 2013. The subjects were firstly screened for presence of GDM, using 50-g glucose challenge test (GCT) at 24-28th weeks of gestation. For the pregnant women with positive results of GCT (plasma glucose ≥140 mg/dl), 100-g three hour oral glucose tolerance test (3 hrs - OGTT) was performed and the subjects with at least two results higher than normal values (fasting < 95 mg/dl, 1 h <180 mg/dl, 2 h <155 mg/dl and 3 < 140 mg/dl ) were diagnosed as GDM [22]. Seventy healthy pregnant women and seventy five pregnant women who had GDM were enrolled in this study. The absence of family history for the T2D, absence of clinical evidence of any major disease and absence of medication use that may alter glucose tolerance were the inclusion criteria for the control pregnant women. The inclusion criteria for the pregnant women with GDM were: (1) newly diagnosed cases, and (2) no previous use of oral hypoglycemic agents. The exclusion criteria from the study were the presence of type-1 or type-2 diabetes mellitus and other known major diseases. Maternal BMI (kg/m^2^) was calculated as the ratio of the weight (kg) to the square of the height (m). The study protocol was approved by the ethics committee at Shiraz university of Medical Sciences, Shiraz, Iran. Informed written consent was obtained from all participants.

### Biochemical determinations

Fasting venous maternal blood sample was collected in both groups, after an overnight 12 hour fasting. Sera were separated immediately and stored at −70 C until biochemical analyses were performed. Fasting plasma glucose (FPG), total cholesterol (TC), triglyceride (TG) and high-density lipoprotein cholesterol (HDL-C) were measured by using commercially available kits. Fasting plasma low-density lipoprotein cholesterol (LDL C) was calculated using the formula of Friedewald et al. [23]; LDL-C = total cholesterol (TC) – (HDL-C) - [triglycerides (TG) ÷ 5]. HbA1C was measured by ion-exchange high performance liquid chromatography. Serum insulin levels were measured by radioimmunoassay using available commercial kits. Serum resistin concentration was measured by immunoassay using a commercially human resistin ELISA kit (Biovendor, Czech Republic) according to the manufacturer’s instructions. The lowest detectable level of serum resistin was 0.012 ng/ml and intra- and inter-assay coefficients of variation of the assay were 5.9% and 7.6%, respectively. In this study, HOMA-IR (homeostasis model of assessment for insulin resistance) was used for evaluation of insulin sensitivity. HOMA-IR is defined as follows: ([fasting glucose] × [fasting insulin])/22.5 [24].

### RETEN gene SNP −420 C/G genotyping

**Figure 1:**
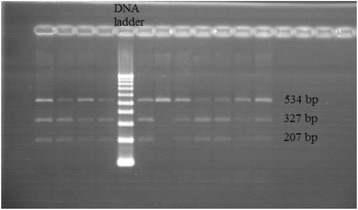
Genotypes of −420 C > G polymorphism of resistin gene determined by PCR-RFLP method and analyzed by a 2% agarose gel electrophoresis stained with ethidium bromide and viewed under UV light. The size of the restriction fragments is shown.

Genomic DNA was extracted from EDTA treated whole blood using DNP™ kit (Cinnagen, Iran). The extracted genomic DNA was stored at −70 C prior to polymerase chain reaction (PCR). Genotyping for detection of SNP −420 C/G of the RETEN gene was performed using PCR-RFLP (restriction fragment length polymorphisms) method. In brief, a DNA fragment containing the SNP −420 C/G was amplified by 5′TGTCATTCTCACCCAGAGACA-3′ as the forward primer and 5′GGGCTCAGCTAACCAAATC-3′ as the reverse primer [25]. PCR thermal cycling was started with initial denaturation at 95°C for 5 min, followed with 35 cycles of amplification which included denaturation at 95°C for 30 sec, annealing at 60°C for 30 sec, extension at 72°C for 30 sec, and final extension at 72°C for 10 min. PCR products were digested by Bbs I restriction enzyme (Fermentas Life Sciences, Lithuania) and separated on 2% agarose gel electrophoresis. The GG homozygous genotype was marked by a single 534 bp (undigested) fragment. PCR products were cleaved into two fragments of 327 and 207 bp in the presence of CC homozygous genotype and three fragments of 534,327 and 207 bp for GC heterozygous genotype (Figure 1). In order to evaluate the reproducibility of the genotyping procedure, duplicate genotyping reactions were performed for 10% of the samples. Genotyping success rate was >98%.

### Statistical methods

All statistical analyses were performed using SPSS 15.0 software (SPSS Inc., Chicago, IL, USA). The normality of distribution of the continuous variables in groups was assessed by the Shapiro–Wilk test. The normally distributed variables were analyzed applying the Student’s *t*-test, and the variables that did not show a normal distribution were compared using Mann–Whitney *U*-test. The data were presented in mean ± standard deviation (SD) or ratio and percent. Bivariate correlation (computing Pearson^’^s coefficient with their significance levels) between maternal serum resistin levels and maternal blood insulin, FPG, HOMA-IR, BMI, gestational age, maternal age in the GDM and non-GDM subjects was calculated. Multiple linear regression analysis was conducted using the stepwise method to determine which factors were significantly and independently associated with maternal serum resistin. The differences in serum resistin levels between GDM and Non- GDM subjects were analyzed using ANCOVA after adjustment for covariates (maternal age, gestational age, BMI, and HOMA-ir).The Chi-square test was used to compare frequencies of allele and genotypes. To examine the independent association of the SNP -420C > G with GDM, univariate and multivariate logistic regression analysis was conducted. In the regression models, GDM status was chosen as the dependent variable and GG and GC + CC genotypes as the independent variable. Confounding variables including maternal BMI, maternal age, and maternal HOMA-index were later included in the model. For all comparisons, the statistical significance was defined by a P < 0.05.

## Results

### Comparison of baseline characteristics between subjects with and without GDM

The baseline demographic and clinical characteristics of patients with GDM and non-GDM controls are shown in Table 1. As can be seen in Table 1, maternal age, gestational age and BMI of the subjects were similar in the groups. GDM patients had a significantly higher insulin resistance index (as assessed by HOMA-IR) (P < 0.01), fasting blood glucose (P < 0.01) and HbA1C (P < 0.001) than non-GDM subjects. Blood lipid levels were similar in the groups. The mean of serum resistin levels was similar in GDM group (13.3 ± 7.4, ranging from 1.1 to 28.0 ng/ml) and non-GDM subjects (11.4 ± 6.9, ranging from 1.3 to 26.7 ng/ml) (Table 1). Adjustment for maternal age, gestational age, BMI category (normal weight; BMI < 25 and overweight/obesity; BMI ≥ 25 ), and HOMA-IR did not significantly change the results.

**Table 1:** **Demographic characteristics and biochemical measurements in the GDM patients and Non- GDM subjects**

	**GDM group(n = 75)**	**Non-GDM group(n = 70)**	**P values**
**Age(y)**	29.4 ± 4.9	26.7 ± 5.0	0.73
**Gestational age(week)**	29.9 ± 3.4	29.6 ± 3.1	0.60
**BMI(Kg/m** ^**2**^ **)**	28.2 ± 4.5	28.1 ± 4.4	0.94
**FPG(mg/dl)**	87.9 ± 22.4	78.7 ± 11.3	0.002*
**HbA1C (%)**	5.80 ± 0.95	5.08 ± 0.37	< 0.001*
**Insulin(μIU/ml)**	18.2 ± 11.6	17.5 ± 7.4	0.67
**HOMA-IR**	4.6 ± 3.1	3.4 ± 1.6	0.005*
**TG(mg/dl)**	267.3 ± 121.2	267.8 ± 101.9	0.98
**TC(mg/dl )**	230.7 ± 50.9	240.0 ± 51.1	0.27
**HDL-C(mg/dl )**	52.4 ± 13.6	52.6 ± 12.4	0.55
**LDL-C(mg/dl )**	113.6 ± 28.7	118.7 ± 27.6	0.28
**Resistin (ng/ml)**	13.0 ± 6.6	11.4 ± 6.9	0.36

GDM: gestational diabetes mellitus, BMI: body mass index. FPG: fasting plasma glucose. HbA1c: hemoglobin A1c. HDL-C: high-density lipoprotein cholesterol. LDL-C: low-density lipoprotein cholesterol. HOMA-IR: homeostasis model assessment index for insulin resistance. Values are presented as mean ± SD. Student- *t* test was used for comparing the data between groups. *Statistically significant.

### Correlation between serum resistin and clinical parameters

Serum resistin concentration did not show any significant correlation with GDM related risk factors including maternal age(r = 0.053; *p* = 522), BMI(r = 0.051; *p* < 0.538), and HOMA-IR(r = 0.006; *p* < 0.946), in entire cohort. Pearson correlation analysis showed that fasting serum resistin concentrations were positively correlated with TG (r = 0.293; *p* < 0.03), TC(*r* = 0.288; *p* = 0.032), LDL-C (*r* = 0.353; *p* = 0.011) in GDM patients. Stepwise linear regression analysis revealed that TG (β = 0.353, P = 0.002) was the independent predictor of serum resistin and accounted for 12.5% of the variance.

### Genotype and allele analysis of SNP -420C > G

The genotype and allele frequencies in our case–control samples are shown in Table 2. The results showed that the frequency of GG genotype (p = 0.025, odds ratio = 2.62, 95% CI; 1.08-6.36) and G allele (p = 0.045, odds ratio = 2.14, 95% CI 0.97-4.73) were higher in the GDM patients compared to the non-GDM group (Table 2). Logistic regression analysis was performed to test the association of SNP -420C > G with GDM. The findings of this analysis showed that the frequency of GG genotype of SNP -420C > G was 3.9-fold higher in GDM women than non-GDM women (odds ratio = 3.9, 95% CI; 1.77-8.56, p = 0.001). To examine the independent association of genotype with susceptibility to GDM, multivariate logistic regression analysis with confounding factors including age, BMI and HOMA-index of mothers, was then conducted. The results (Table 3) showed that after adjusting for covariates, the frequency of GG genotype of SNP -420C > G was still higher in GDM women than non-GDM women (odds ratio = 4.59, 95% CI; 1.96-10.71, p = 0.00). There were no significant differences between genotypes of SNP - 420C > G with respect to BMI and circulating concentrations of serum resistin (Table 4).

**Table 2:** **Genotype and allele frequencies of RETN gene SNP −420 C/G in the non – GDM (control) and GDM patients**

		**Genotype frequency**			**Allele frequency**		
**Group**	**n**	**GG**	**GC + CC**	**p**	**G**	**C**	**p**
**Non-GDM**	67	11(16.4%)	56(83.6%)	0.025*	69(51.5%)	65(49.5%)	0.045*
**GDM**	75	33(44%)	42(56%)		108(72%)	42(28%)	
		OR (95% CI), 4.0 (1. 8–8.8)			OR (95% CI), 2.4 (1.48 -3.96)		

Genotypes are reported as number with percent in parentheses. *Two**-**tailed chi-square test (χ^2^). 95% CI = 95% confidence interval.

**Table 3:** **Multivariate logistic regression analysis for gestational diabetes**

**Variables**	**B**	**SE**	**Wald**	**P**	**OR**	**95.0% C.I**
Age	0.13	0.04	9.27	0.001	1.138	1.05-1.23
SNP - 420 C/G	1.52	0.46	12.41	0.000	4.59	1.96-10.71
HOMA-IR	0.18	0.11	2.62	0.156	1.177	0.94-1.47
BMI	0.03	0.07	0.01	0.642	1.031	0.91-1.17

Logistic regression analysis was performed for SNP - 420 C/G with adjustment for confounding factors (age, BMI, and HOMA-IR). BMI: body mass index, HOMA-IR: homeostasis model assessment index for insulin resistance. OR; odds ratios, CI; confidence intervals.

**Table 4:** **Variations in serum resistin concentration (ng/ml) and BMI (kg/mg** ^**2**^ **) by genotypes of the SNP −420 C/G**

**Variable**	**GDM patients genotype**			**Non-GDM subjects genotype**		
	**GG**	**GC + CC**	**P value**	**GG**	**GC + CC**	**P value**
**Resistin (ng/ml)**	13.8 ± 5.3	12.5 ± 7.2	0.41	13.7 ± 2.9	11.9 ± 7.7	0.43
	(11.7- 15.9)	(10.3 - 14.7)		(11.8- 15.5)	(9.8 - 13.9)	
**BMI(Kg/m** ^**2**^ **)**	28.2 ± 5.3	28.2 ± 3.3	0.95	28.2 ± 4.4	27.9 ± 4.1	0.84
	(26.6 – 29.9)	(27.0 – 29.4)		(27.0 – 29.3)	(25.3 – 30.6)	

Values are mean ± SD (95% confidence interval). ANOVA and least significant difference (LSD) as post hoc tests was used to compare the means of circulating resistin and BMI between different genotype of SNP -420C/G. BMI: body mass index.

## Discussion

The findings of this study showed that GG genotype and G- allele of SNP- 420C/G in the RETN gene is associated with genetic susceptibility to GDM in our population. The second finding of this study was the positive correlation between the serum concentration of atherogenic lipids and serum resistin. However, no significant associations were observed between serum resistin levels, SNP- 420C/G in the patients with GDM.

Allelic and genotypic frequencies of SNP- 420C/G (rs1862513) show ethnic variation [21]. In this study, the frequency of rare G/G genotypes and G allele among the control NGT women was 16.4% and 51.5%, respectively. This is closely comparable with another Iranian data by Emamogolipour, et al. [26] but higher than those reported for healthy Caucasian, Chinese, Korean and Japanese subject (24% to 30%) [18,27,28]. Previous studies have shown an association between G allele of SNP- 420C/G and T2D in Iranian and other population [19,26]. To best of our knowledge, this is the first study that revealed the association of SNP- 420C/G with GDM. Our data showed that the frequency of GG genotype and G allele was significantly higher in the GDM patients compared to normal pregnant group. The exact molecular mechanisms that link this polymorphism with GDM are not known at present. It has been previously reported that G allele of SNP- 420 in the promoter of RETN gene was associated with increase in circulating resistin [18,19]. According to the authors, this could be related to specific recognition of – 420G by Sp 1/3, which increases RETN promoter activity, leading to enhanced serum resistin levels. In this study, we did not observe any association between serum resistin levels and SNP- 420G. It has been reported that resistin gene expression is controlled by genetics and environmental factors such as dietary habits [29,30]. Indeed, an ethnic specific resistin gene expression has been suggested [31]. Onum et al. [32] suggested that this ethnic difference may be related to SNP-385 in the RETN gene. They showed that combination of A allele of SNP- 358 with the G allele of SNP-420 in subjects with G-A haplotype conferred the highest circulating resistin concentration. On the basis of onum et al.’s hypothesis, the ethnic differences in SNP- 358 might account for the discrepancy observed in association of SNP- 420 with serum resistin in different population [32]. We did not evaluate SNP-358 in this study; hence, it is not clear whether the A of SNP- 358 indeed accounts for of any association between plasma rsistin and SPN-420 in our population.

In agreement with the results of two previous studies [33,34], our findings showed that serum resistin levels were similar in the GDM and non- GDM subjects. By contrast, other studies have found lower [7] or higher [15,16] resistin levels in GDM patients compared to healthy pregnant women. These discrepancies mostly arise from the presence of heterogeneities in the several aspects of aforementioned studies. One of the causes of these heterogeneities among studies is the application of different GDM diagnostic methods, i.e., National Diabetes Data Group (NDDG, 100 g 3 hrs OGTT), Carpenter and Coustan (100 g 3 hrs OGTT), and WHO criteria (75 g 2 hrs OGTT) [35]. Since these methods had different criteria for diagnosis of GDM, the population of GDM patients as well as control subjects that enrolled in the aforementioned studies substantially different in aspect of level of hyperglycemia and severity of GDM. Moreover, it has been reported that the maternal serum resisin is increased in concomitant with the increase in the gestational age [36]. Therefore, difference in the gestational age of the subjects at the time blood sampling for resistin assay is the second cause of heterogeneity between these studies. The discrepancies between the studies may also be a reflection of genetic and anthropometric variations among ethnic groups that enrolled in these studies, as some functional polymorphism in the resistin gene as well as obesity has been showed that influenced on the expression of resistin in some ethnic group. Finally, difference in preparation of sample (serum/plasma), conditions of sample storage, and assay methods which used for the quantification of resistin may also consider as a plausible explanation for difference among studies, as variable results has been reported for the concentration circulating resistin in these studies [35].

Resistin has been reported to be related to obesity and insulin in patients with T2D [20,37]. Similar to T2D insulin resistance and obesity are involved in the etiology of GDM [2]. Increases in HOMA-ir and serum insulin concentration are the representative parameter of insulin resistance [38]. Consistent with other studies, the results of the present study revealed that HOMA- ir was significantly higher in GDM patients compared to healthy pregnant women. However, serum resistin levels in GDM group did not show any correlation with HOMA-ir, serum insulin concentration. These results are in line with other studies [13,34], suggesting that serum resistin levels may not be associated with insulin resistance in GMD patients.

The increase in serum TG, total cholesterol, LDL cholesterol concentration and high TG/HDL-C ratio are atherogenic markers that correlate with insulin resistance [39]. Similar to other studies [30,40], a strong positive correlation was found among serum resistin, serum TG, total cholesterol, LDL cholesterol concentration, and TG/ HDL ratio in the present study. Our results also demonstrated that the serum levels of atherogenic lipids were elevated in GDM patients with the highest quartile resistin, which suggested that increase in resistin concentration might be closely associated with modulation of lipid metabolism and may play a role in dyslipidemia in GDM patients.

Several SNPs were known in the RETN gene but we considered only the SNP-420C > G in the promoter of gene because of its possible influence on RETN gene expression and circulating resistin concentration. Hence, further studies will be required to clarify the association among resistin gene polymorphisms, circulating resistin and GDM.

## Conclusion

In conclusion, the findings of the present study suggest that GG genotype of SNP- 420C/G in the promoter of RETN associated with genetic susceptibility to GDM in our population. However, we failed to show any association between this polymorphism and known risk factors of GDM. Thus, further studies are necessary to confirm the role of this polymorphism in the pathogenesis of GDM and to explore the potential mechanisms by which it modulates susceptibility to GDM.
